# Cortisol overproduction results from DNA methylation of CYP11B1 in hypercortisolemia

**DOI:** 10.1038/s41598-017-11435-2

**Published:** 2017-09-11

**Authors:** Mitsuhiro Kometani, Takashi Yoneda, Masashi Demura, Hiroshi Koide, Koshiro Nishimoto, Kuniaki Mukai, Celso E. Gomez-Sanchez, Tadayuki Akagi, Takashi Yokota, Shin-ichi Horike, Shigehiro Karashima, Isamu Miyamori, Masakazu Yamagishi, Yoshiyu Takeda

**Affiliations:** 1 Division of Endocrinology and Hypertension, Department of Cardiovascular and Internal Medicine, Kanazawa University Graduate School of Medicine, Kanazawa, Ishikawa, 920-8640 Japan; 2 Program Management Office for Paradigms Establishing Centers for Fostering Medical Researchers of the Future, Kanazawa University, Kanazawa, Ishikawa, 920-8640 Japan; 3 Department of Hygiene, Kanazawa University Graduate School of Medicine, Kanazawa, Ishikawa, 920-8641 Japan; 4 Laboratory of Molecular and Biochemical Research, Research Support Center, Juntendo University Graduate School of Medicine, Tokyo, 113-8421 Japan; 5 Department of Uro-Oncology, Saitama Medical University International Medical Center, Hidaka, Saitama, 350-1241 Japan; 6 Department of Biochemistry and Medical Education Center, Keio University School of Medicine, Tokyo, 160-8582 Japan; 7 Endocrinology Section, G.V. (Sonny) Montgomery VA Medical Center and University of Mississippi Medical Center, Jackson, MS 39216 USA; 8 Department of Stem Cell Biology, Graduate School of Medical Sciences, Kanazawa University, Kanazawa, Ishikawa, 920-8640 Japan; 9 Advanced Science Research Center, Kanazawa University, Kanazawa, Ishikawa, 920-8640 Japan; 10 University of Fukui, Yoshida-gun, Fukui, 910-1193 Japan

## Abstract

Adrenocortical hormone excess, due to primary aldosteronism (PA) or hypercortisolemia, causes hypertension and cardiovascular complications. In PA, hypomethylation of aldosterone synthase (*CYP11B2*) is associated with aldosterone overproduction. However, in hypercortisolemia, the role of DNA methylation of 11β-hydroxylase (*CYP11B1*), which catalyzes cortisol biosynthesis and is highly homologous to *CYP11B2*, is unclear. The aims of our study were to determine whether the *CYP11B1* expression was regulated through DNA methylation in hypercortisolemia with cortisol-producing adenoma (CPA), and to investigate a possible relationship between DNA methylation and somatic mutations identified in CPA. Methylation analysis showed that the *CYP11B1* promoter was significantly less methylated in CPA than in adjacent unaffected adrenal tissue and white blood cells. Furthermore, in CPA with somatic mutations in either the catalytic subunit of protein kinase A (*PRKACA*) or the guanine nucleotide-binding protein subunit alpha (*GNAS*) gene, the *CYP11B1* promoter was significantly hypomethylated. In addition, DNA methylation reduced *CYP11B1* promoter activity using a reporter assay. Our study results suggest that DNA methylation at the *CYP11B1* promoter plays a role in the regulation of *CYP11B1* expression and cortisol production in CPA, and that somatic mutations associated with CPA reduce DNA methylation at the *CYP11B1* promoter.

## Introduction

Hypercortisolemia is a hormonal disorder caused by the prolonged exposure of the body to high levels of cortisol. Cortisol excess in hypercortisolemia is associated with substantial morbidity and mortality. Metabolic disorders such as hypertension, diabetes mellitus, and hyperlipidemia are common among hypercortisolemia patients and contribute to increased cardiovascular complications^1^. Endogenous hypercortisolemia results from a variety of diseases and disorders, including cortisol-producing adenoma (CPA), adrenal carcinoma, primary pigmented nodular adrenocortical disease (PPNAD), bilateral adrenal hyperplasia (BAH), adrenocorticotropic hormone (ACTH)-independent macronodular adrenocortical hyperplasia (AIMAH), excess ACTH produced by the pituitary (Cushing’s disease) or by ectopic tumors producing ACTH (ectopic Cushing’s syndrome)^2^.

Cortisol biosynthesis is mainly regulated by the cyclic AMP (cAMP)/protein kinase A (PKA) signaling pathway activated by ACTH secreted from the anterior pituitary gland^3^. In this pathway, 11β-hydroxylase (cytochrome P450 family 11 subfamily B member 1: *CYP11B1*) catalyzes the final step of cortisol biosynthesis. Recently, exome sequencing revealed that CPAs frequently carry somatic mutations in the catalytic subunit of PKA (*PRKACA*) that leads to the activation of cAMP response element-binding protein (CREB), and the guanine nucleotide-binding protein subunit alpha (*GNAS*) gene, resulting in an activation of PKA^4, 5^. Both mutations result in increase of *CYP11B1* expression and thereby an excessive production of cortisol. However, the molecular mechanism how these mutations affect *CYP11B1* expression has not been well clarified.

DNA methylation is a fundamental epigenetic mechanism that regulates gene expression^6^. Generally, gene transcription is active at unmethylated DNA regions, and DNA methylation results in reduced gene expression. Recent studies demonstrated that aldosterone synthase (cytochrome P450 family 11 subfamily B member 2: *CYP11B2*) in aldosterone-producing adenomas (APA) is upregulated by DNA hypomethylation of its promoter region^7–9^. Aldosterone is secreted by zona glomerulosa, the outermost layer in the adrenal cortex, and cortisol is secreted by zona fasciculata, which is situated between the glomerulosa and reticularis. In addition, *CYP11B2* shows high homology to the *CYP11B1*, and both are located on chromosome 8 (8q24.3)^10^. However, the relationship between *CYP11B1* overexpression and DNA methylation in hypercortisolemia has yet to be elucidated. Therefore, it is intriguing to focus on CPA that overexpresses *CYP11B1* ^11, 12^.

In this study, to clarify the molecular mechanism of cortisol production in hypercortisolemia, we examined whether the CYP11B1 expression in CPA was regulated through DNA methylation by carrying out multiple experiments.

## Results

### CYP11B1 expression in cortisol-producing adenomas

**Figure 1:**
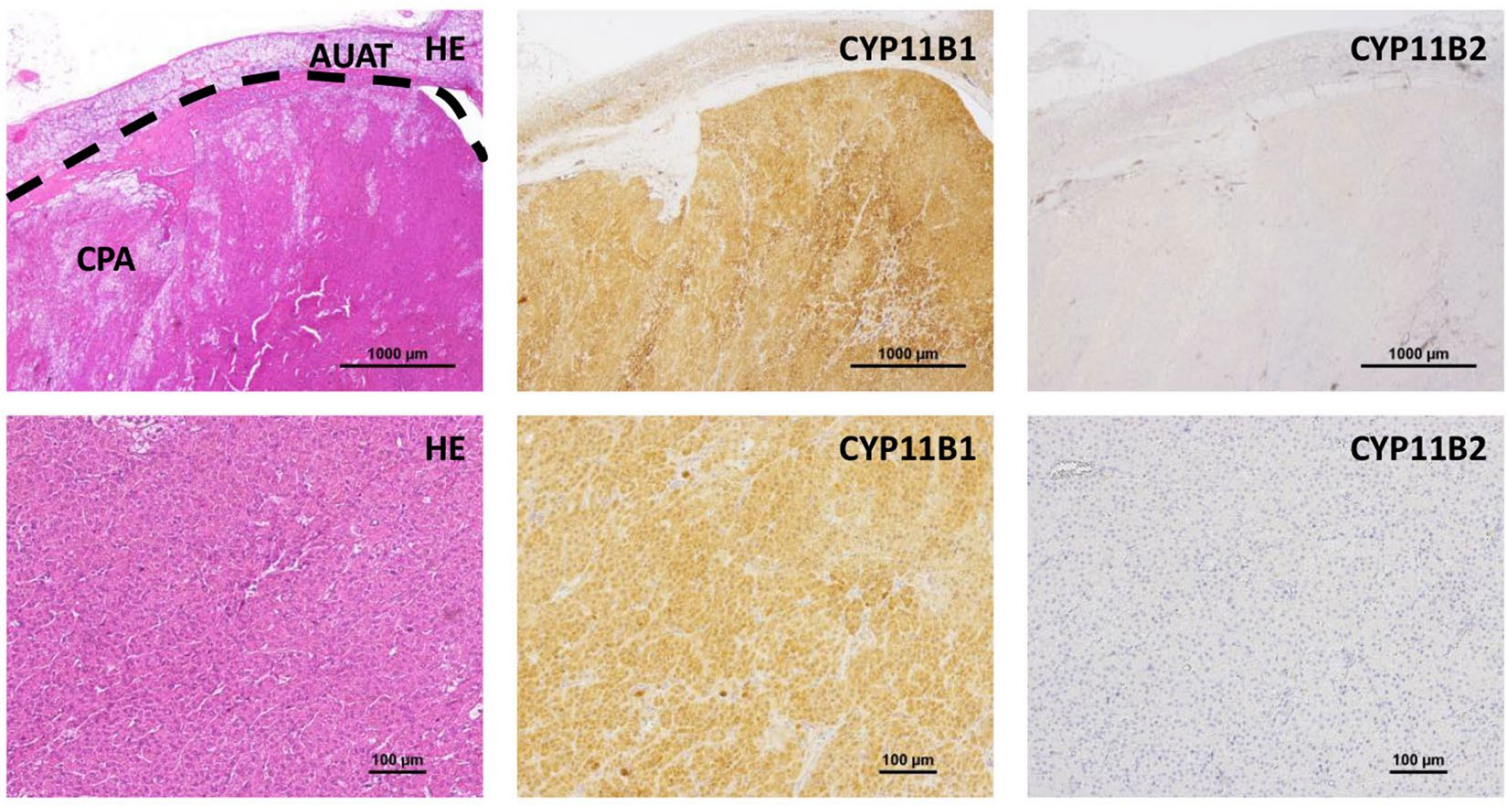
Confirmation of *CYP11B1* overexpression in cortisol-producing adenomas. The immunohistochemical analysis of case #1 is shown as representative of all 13 cases. Formalin-fixed paraffin-embedded tissue sections were stained with Hematoxylin & Eosin (HE), anti-CYP11B1, and anti-CYP11B2 antibodies. CPA, cortisol-producing adenoma; AUAT, adjacent unaffected adrenal tissue.

Immunohistochemical analysis revealed that all CPAs showed positive staining for CYP11B1 while staining negative for aldosterone synthase, CYP11B2 (Fig. 1, Figure S1). Patient 10 had two adenomas with different staining patterns (Figure S2), the adenoma with stronger staining for CYP11B1 was defined as the culprit lesion and used in the following experiments. We next compared the expression level of *CYP11B1* between CPA and adjacent unaffected adrenal tissue (AUAT), and found that CPA expresses *CYP11B1* at higher level than AUAT (Figure S3). In addition, we performed Western blot analysis and confirmed that CYP11B1 protein level was significantly higher in CPA than in AUAT (Figure S4).

### Prevalence of *PRKACA* or *GNAS* gene mutations in cortisol-producing adenomas

Eight of the 13 CPA patients had somatic mutations in either the *PRKACA* or the *GNAS* gene (Table 1, Table S1, Figure S5). Two patients had the p.L206R mutation of the *PRKACA* gene. Among six patients with *GNAS* gene mutations, three CPAs had p.R201H. The other three patients carried p.R201C, p.R201S, and p.Q227R mutations respectively. None of the patients had somatic mutations in *catenin beta-1* (*CTNNB1*) gene. There was no significant difference in the clinical characteristics of patients with and without mutations (Table 1).

**Table 1:** Summary of clinical characteristics of patients with cortisol-producing adenomas in this study.

	All patients	CPA with *PRKACA* or *GNAS* mutation	CPA without *PRKACA* or *GNAS* mutation
Number of cases, n	13	8	5
Age, y [mean (range)]	53 (33–65)	52 (37–64)	53 (33−65)
Sex, males/females	4/9	2/6	2/3
Body mass index, kg/m^2^	24 ± 4	24 ± 2	25 ± 6
Systolic blood pressure, mmHg	146 ± 25	150 ± 9	139 ± 2
Diastolic blood pressure, mmHg	90 ± 15	93 ± 6	85 ± 3
Serum potassium, mEq/L	3.8 ± 0.3	3.7 ± 0.1	3.8 ± 0.2
Baseline F, nmol/L	362 ± 31	373 ± 41	343 ± 50
24 h urinary F, µg/day	58 ± 10^†^	64 ± 15^‡^	46 ± 8^§^
Midnight F, nmol/L	268 ± 50^†^	255 ± 61^‡^	290 ± 98^§^
F after 1 mg DST, nmol/L	241 ± 51	249 ± 55	227 ± 110
Adrenal tumor size, mm	26 ± 2	23 ± 2	30 ± 4

^†^n = 11, ^‡^n = 7, ^§^n = 4. Data presented as mean ± SEM, except where noted otherwise.

F, serum cortisol concentration; DST, dexamethasone suppression test.

### CpG sites and transcription factor-binding sites of the *CYP11B1* promoter in cortisol-producing adenoma

**Figure 2:**
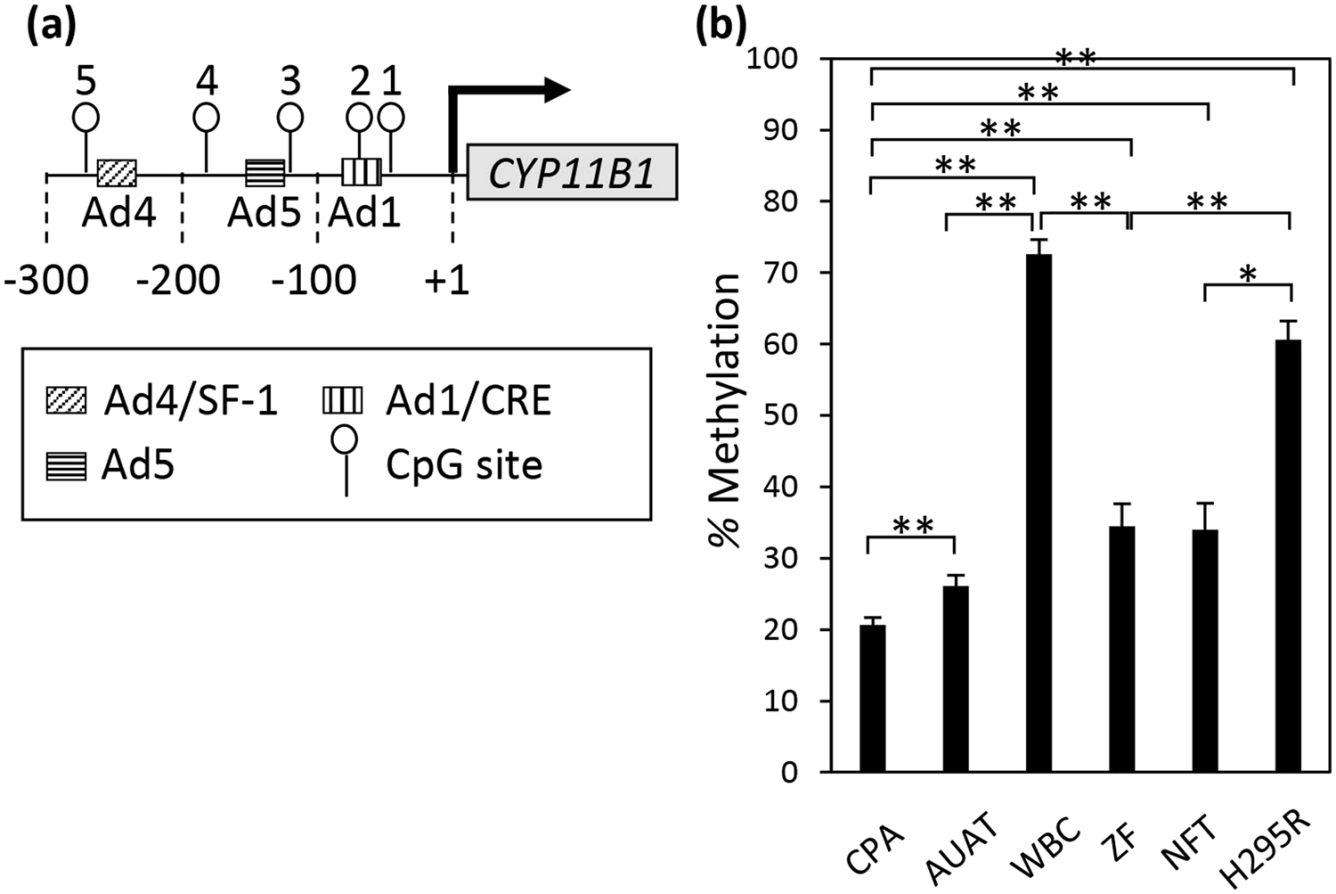
Hypomethylation of the *CYP11B1* promoter in cortisol-producing adenomas. (**a**) CpG sites and transcription factor-binding sites in the human *CYP11B1* promoter. Nucleotide numbers are relative to the transcription start site. CpG sites around the transcription factor-binding sites are denoted as lollipops and numbered. b, Comparison of methylation levels of the *CYP11B1* promoter among cortisol-producing adenomas (CPA) (n = 13), adjacent unaffected adrenal tissue (AUAT) (n = 13), white blood cells (WBC) (n = 13), zona fasciculata (ZF) of normal adrenal cortex (n = 7), non-functioning adrenal tumor (NFT) (n = 7), and H295R cells (H295R) (n = 13). Methylation levels at five CpG sites were measured by pyrosequencing. Data are shown as the mean ± SEM, and analyzed with the Mann-Whitney U test between each two groups. *P < 0.05, **P < 0.01.

The CpG site is a DNA region where a cytosine nucleotide occurs next to a guanine nucleotide in the linear sequence of genome DNA, and is a target for DNA methylation. DNA methylation occurs almost exclusively at cytosine of CpG site. When we manually searched for CpG sites in the *CYP11B1* promoter region, we found five CpG sites, and all of them are present near transcription factor-binding sites (Fig. 2a, Figure S6).

### Methylation status of the *CYP11B1* promoter in cortisol-producing adenoma

**Figure 3:**
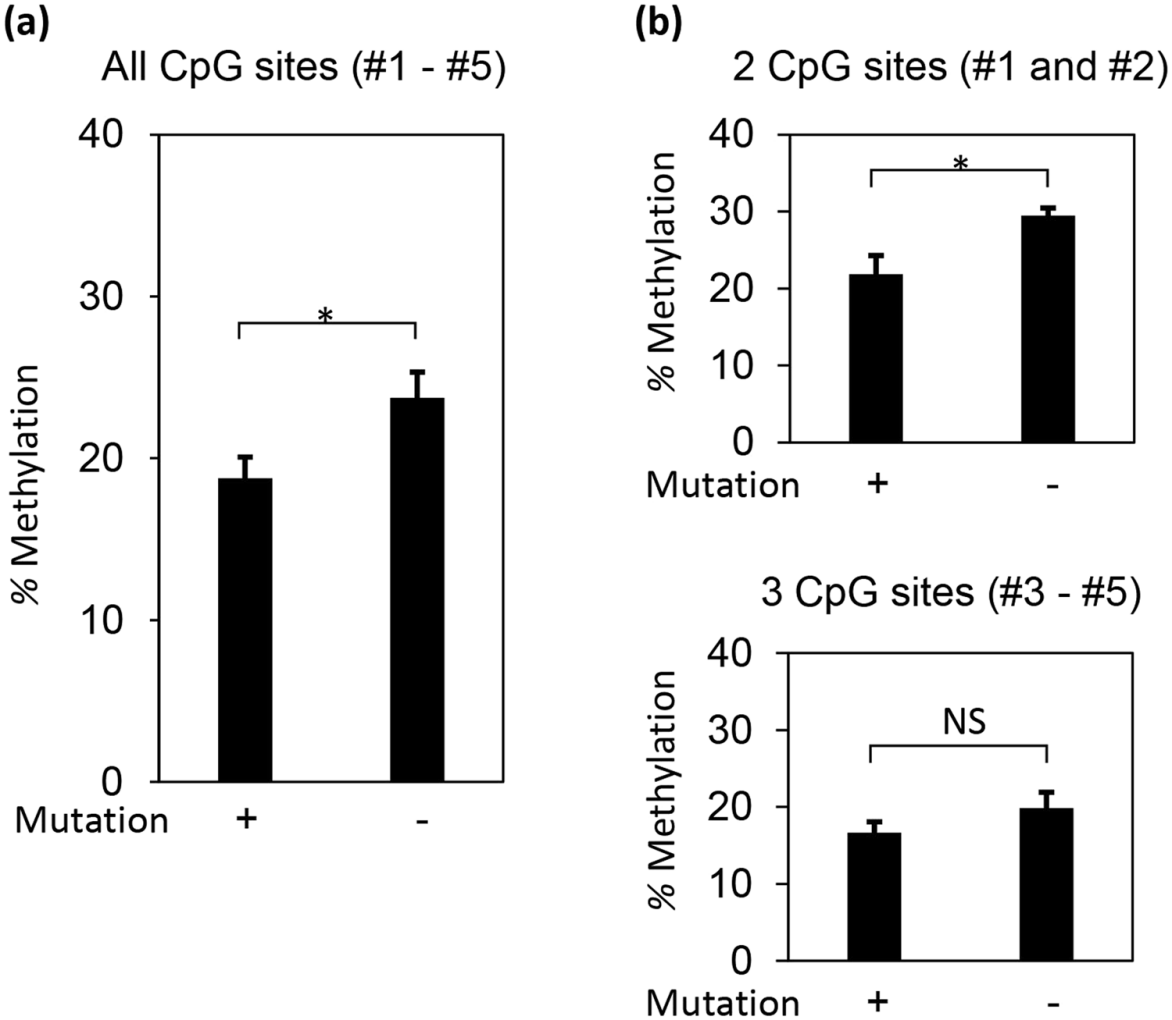
*PRKACA* or *GNAS* mutations induce hypomethylation of the *CYP11B1* promoter in cortisol-producing adenomas. Methylation levels were compared between cortisol-producing adenoma with (+) and without (−) mutations at all five CpG sites (**a**), two CpG sites around the Ad1/CRE binding site (b, upper panel) and the other three CpG sites (b, lower panel). Data are shown as the mean ± SEM (n = 8 for with mutations, n = 5 for without mutations), and analyzed with the Mann-Whitney U test. *P < 0.05. NS, not significant.

To examine the role of DNA methylation on high *CYP11B1* expression in CPA, we compared the DNA methylation level of five CpG sites in the *CYP11B1* promoter (Fig. 2a, Figure S6) in CPA with the same sites in AUAT, white blood cells (WBC), zona fasciculata (ZF) of normal adrenal cortex, non-functioning adrenal tumor (NFT) and H295R cells. The methylation of all five sites in CPA was significantly lower than in AUAT, WBC, ZF, NFT and H295R cells (Fig. 2b). Furthermore, methylation status was also significantly lower in AUAT, ZF and NFT, compared to WBC and H295R cells. Additionally, CPAs that carried somatic mutations (n = 8) were significantly less methylated in the five CpG sites of the *CYP11B1* promoter compared to those without mutations (n = 5) (Fig. 3a).

Mutations in both *PRKACA* and *GNAS* facilitate phosphorylation of the transcription factor CREB^5^, which then binds to the Ad1/ cAMP response element (CRE) site in the *CYP11B1* promoter. Therefore, we focused our analysis on the two CpG sites (CpG#1 and CpG#2 in Fig. 2a) near the Ad1/CRE site. In CPA, the somatic mutations reduced the methylation level of these two CpG sites significantly, while the methylation level of the other three CpG sites were not affected by somatic mutations (Fig. 3b).

### *CYP11B1* promoter activity is regulated by DNA methylation

**Figure 4:**
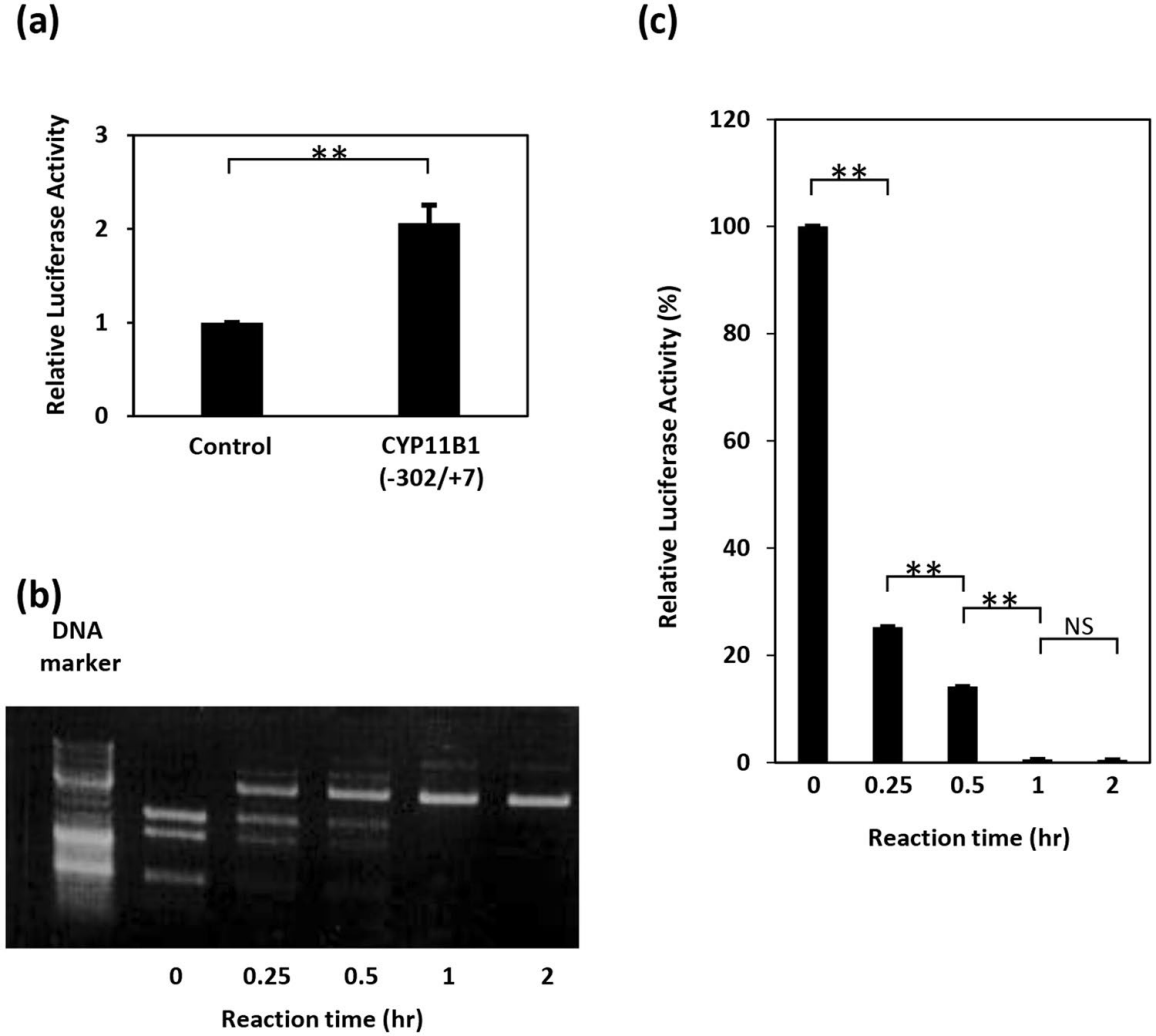
DNA methylation suppresses *CYP11B1* promoter activity. (**a**) Confirmation of *CYP11B1* promoter activity. H295R cells were transiently transfected with pGL4.10[luc2] (control) or pGL4-cyp11b1[−302/+7](CYP11B1(−302/+7)). Two days after transfection, cells were lysed and luciferase activity was measured. The luciferase activity of the control sample was set to 1.0, and data are shown as the mean ± SEM (n = 6), and analyzed with the Mann-Whitney U test. **P < 0.01. (**b**) Confirmation of plasmid methylation. The *CYP11B1* reporter plasmids were incubated with the CpG methyltransferase M.SssI for the indicated periods. The methylated plasmids were then digested by the methylation-sensitive restriction enzyme BsiEI and subjected to agarose electrophoresis. Note that BsiE1 cannot digest methylated plasmids. Data shown are representative of three independent experiments. Full-length gel is presented in Supplementary Figure S9. (**c**) Methylation suppresses *CYP11B1* promoter activity. After incubation with M.SssI for the indicated periods, the *CYP11B1* reporter plasmids were transfected into H295R cells, whose lysates were subjected to luciferase assay. Luciferase activity of the control sample (reaction time = 0 h) was set to 100%, and the mean ± SEM (n = 4) of the data are shown. Data are analyzed with the Mann-Whitney U test. *P < 0.05. NS, not significant.

We examined the *CYP11B1* promoter activity using a luciferase assay. We isolated the *CYP11B1* promoter region (−302/+7) that contains five CpG sites around three transcription factor-binding sites: Ad4/steroidogenic factor (SF)−1, Ad5, and Ad1/CRE (Fig. 2a). The *CYP11B1* promoter showed significant activity (Fig. 4a) when a reporter plasmid carrying this promoter region was transfected into human adrenocortical carcinoma H295R cells.

To evaluate the effect of DNA methylation on *CYP11B1* promoter activity, the reporter plasmid was methylated *in vitro* by the CpG-specific methyltransferase M.SssI. The methylation level was confirmed by digestion with the methylation-sensitive restriction enzyme BsiEI (Fig. 4b). When the methylated plasmids were transfected into H295R cells, the promoter activity was reduced proportionally with the increase in DNA methylation (Fig. 4c, Figure S7).

### DNA hypomethylation around the Ad1/CRE site is induced by cyclic AMP stimulation

**Figure 5:**
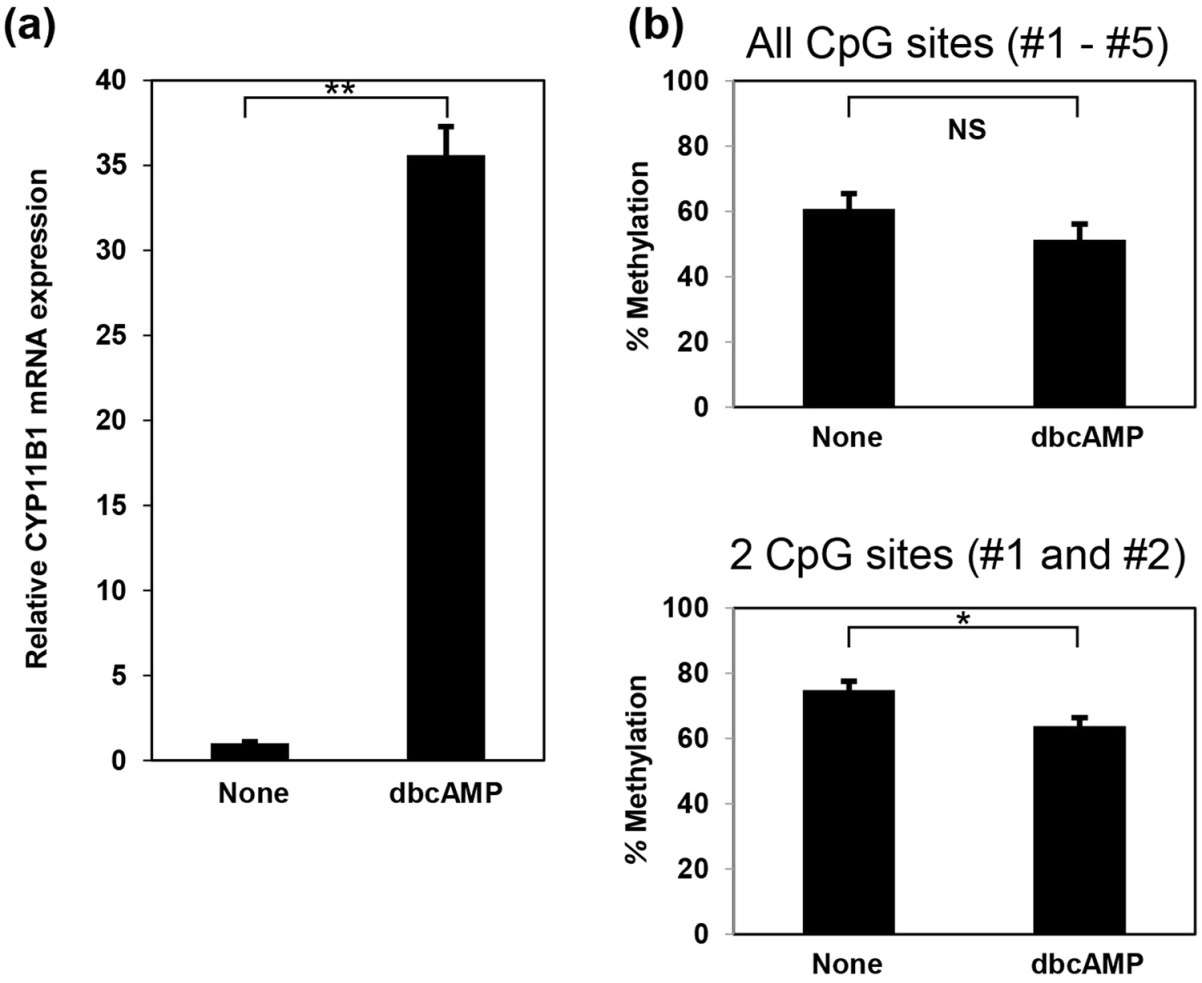
Activation of cAMP signaling results in hypomethylation of the *CYP11B1* promoter. (**a**) dbcAMP stimulation increases *CYP11B1* expression. H295R cells were stimulated with 125 µmol/L dbcAMP for three days, and then the expression level of *CYP11B1* was examined with real-time RT-PCR analysis. The value of no stimulation (None) was set to 1.0, and data are shown as the mean ± SEM (n = 6) and analyzed with the Mann-Whitney U test. **P < 0.01. (**b**) dbcAMP treatment reduces methylation in the *CYP11B1* promoter. After stimulation with dbcAMP, methylation levels at all five CpG sites (upper panel) and two CpG sites around the Ad1/CRE binding site (lower panel) in the *CYP11B1* promoter were measured by pyrosequencing. In both experiments, data are shown as the mean ± SEM (n = 3), and analyzed with the Mann-Whitney U test. NS, not significant. *P < 0.05.

Since mutations in both *PRKACA* and *GNAS* genes lead to the constitutive activation of the PKA pathway, we examined the effect of cAMP-induced PKA activation on DNA methylation using a cAMP analog, 2′-O-dibutyladenosine 3′, 5′-cyclic monophosphate (dibutyric cAMP; dbcAMP). When we treated H295R cells with dbcAMP, the level of *CYP11B1* mRNA significantly increased (Fig. 5a). Furthermore, dbcAMP stimulation significantly reduced DNA methylation at only the two CpG sites around the Ad1/CRE site (Fig. 5b, lower panel). We did not observe significant reduction in methylation levels in the other CpG sites (Fig. 5b, upper panel, Figure S8).

## Discussion

In the present study, we found that the *CYP11B1* promoter in CPA is significantly hypomethylated compared to those in AUAT and ZF. Furthermore, the experiments using H295R cells demonstrated that DNA methylation negatively regulated *CYP11B1* promoter activity. These results demonstrated that DNA methylation is involved in the regulation of *CYP11B1* expression, and plays an important role in *CYP11B1* overexpression in CPA. To our knowledge, this is the first study showing that DNA methylation of the *CYP11B1* promoter is associated with cortisol overproduction in CPA.

In DNA methylation analysis, we compared the methylation status of *CYP11B1* among CPA, AUAT, WBC, ZF, NFT, and H295R cells. Although *CYP11B1* is not expressed in WBC, we performed this comparison because epigenetic changes could be mediated by various stimuli, thus making it important to demonstrate the methylation change observed in CPA (hypomethylation of *CYP11B1*) was not nonspecific^6, 13^. We also used H295R cells as a control because this cell line has the ability to produce mainly not cortisol but adrenal androgens under normal conditions.

DNA methylation regulates gene expression by blocking the binding of transcription factors to DNA^13^. When a cytosine residue is methylated at a CpG site, methyl-CpG-binding domain proteins that promote the formation of transcriptionally inactive forms of chromatin are recruited^5^. Transcription factors mainly bind to promoter regions. Recent studies have reported that the *CYP11B2* promoter in APA is hypomethylated, suggesting that the hypomethylated state is associated with aldosterone overproduction^7–9^. To clarify the relationship between DNA methylation and the promoter activity, we performed experiments using H295R cells.

*CYP11B1* (11β-hydroxylase) and *CYP11B2* (aldosterone synthase) are highly homologous genes^10^ and have three kinds of transcription factor-binding sites in their promoter regions: Ad1/CRE, Ad5, and Ad4/SF-1^14^. The Ad1/CRE site has been shown to play a critical role in the transcription of *CYP11B1* and is recognized by CREB, activating transfactor-1 (ATF-1) and ATF-2^15^. Furthermore, a recent report demonstrated that the Ad5 and Ad4/SF-1 sites, which are binding sites for estrogen-related receptor-α and SF-1 respectively, also play a role in the regulation of *CYP11B1* expression^14^. In the present study, we identified five CpG sites in the *CYP11B1* promoter, all of which were present near transcription factor-binding sites. DNA methylation in the *CYP11B1* promoter reduced its activity. We therefore conclude that DNA methylation interferes with the binding of transcription factors to the *CYP11B1* promoter.

Recent genetic analysis identified a mutation in the *PRKACA* gene that causes CPA^4^. This mutation results in the release of PRKACA from the inhibitory R subunits, leading to the constitutive, cAMP-independent activation of PKA^5^. Moreover, *GNAS* mutations that activate PKA through a constitutive increase in intracellular cAMP concentrations are also frequently identified in CPA^5, 16^. Thus, both *PRKACA* and *GNAS* mutations lead to activation of the cAMP/PKA signaling pathway. Activated PKA phosphorylates CREB, and activated CREB binds to the Ad1/CRE site in the *CYP11B1* promoter to induce *CYP11B1* expression. A previous study reported that mutations in *PRKACA* and *GNAS* were associated with small tumors, young age at presentation, and a severe phenotype^17^. Another study also reported that patients with CPA containing *PRKACA* mutations had higher basal serum cortisol concentrations than patients without *PRKACA* mutations, which persisted after dexamethasone suppression tests^15^. However, in our study, the presence of somatic mutations made no difference in patients’ clinical characteristics. This might be because our study contained fewer cases than previous studies.

Multiple causal genes of APA have also been newly discovered. Mutations in *KCNJ5* ^18^, *ATP1A1* ^19^, *ATP2B3* ^19^, and *CACNA1D* ^20^ upregulate *CYP11B2*. Previous studies reported that there was no correlation between somatic mutations and DNA methylation in APA^6, 8^. However, in the case of CPA carrying *PRKACA* or *GNAS* mutations *CYP11B1* promoters are significantly hypomethylated compared to wild-type CPA. More interestingly, *PRKACA* or *GNAS* mutations reduced DNA methylation at the two CpG sites around the Ad1/CRE binding site significantly, but not at the other three CpG sites. Since these mutations result in the continuous PKA activation, to mimick the effect of these mutations, we treated H295R cells with dbcAMP, which activates PKA constitutively. Similarly, we observed that short-term stimulation with dbcAMP also led to the demethylation of these two CpG sites, but not the other CpG sites in the promoter. These data suggest that mutations in *PRKACA* or *GNAS* have an impact on the methylation status of the *CYP11B1* promoter region, especially around the Ad1/CRE site. Since several studies have demonstrated that the binding of transcription factors affects DNA methylation status^21–23^, these results suggest that somatic mutations commonly found in CPA that facilitate the binding of activated CREB to the Ad1/CRE site should influence DNA methylation of the *CYP11B1* promoter.

In this study, we used H295R cells to examine the effect of dbcAMP on *CYP11B1* expression. This cell line is derived from adrenal cancer and has the ability to produce adrenal androgens, but not cortisol. To correspond with this, the *CYP11B1* promoter in H295R cells is more methylated than those in CPA and AUAT. Thus, our results regarding dbcAMP-stimulated hypomethylation in H295R cells might not reflect the effect of dbcAMP on CPA. The establishment of cortisol-producing cell line or primary culture from adrenal cortex would clarify the effect of dbcAMP on CPA in future.

DNA methylation might be important to not only disease states, but also to the normal physiological status of the adrenal glands. The adrenal cortex forms a laminar structure^24^ where cortisol is produced by ZF in the middle layer of the adrenal cortex, whereas the zona glomerulosa in the outer layer produces aldosterone. Our methylation analysis revealed that in CPA, the *CYP11B1* promoter is significantly hypomethylated, as is *CYP11B2* in APA^7–9^. These observations suggest that expression of both *CYP11B1* and *CYP11B2* are regulated by DNA methylation in adrenocortical adenomas. This raises the possibility that hypomethylation at the promoter regions of *CYP11B1* and *CYP11B2* might be associated with the production of cortisol in ZF and aldosterone in the zona glomerulosa. In this study, we used the samples from normal adrenal cortex with non-CPA patients in order to examine the methylation status of normal ZF. This is because AUAT with CPA patients had atrophied due to exposure to excess cortisol, and we could not identify the obvious remaining ZF (Figure S3). Our results indicated that the *CYP11B1* promoter in normal ZF was significantly more hypomethylated than that in WBC or H295R cells. However, there was no difference in methylation status between ZF and NFT. Then, there may be the difference by the molecular mechanism unlike DNA methylation among both. It would be interesting to evaluate the methylation levels of the *CYP11B1* and *CYP11B2* promoters in each layer of the adrenal cortex samples in future studies.

In summary, DNA demethylation at the promoter region of the *CYP11B1* gene plays an important role in the production of excess cortisol in CPA. Furthermore, somatic mutations associated with CPA, which result in the activation of the cAMP/PKA signaling pathway, induce DNA hypomethylation at the *CYP11B1* promoter. Methylation analysis of the promoter of key enzymes appears to play a significant role in the expression of the enzymes and physiological regulation of hormone biosynthesis. Therefore, DNA methylation at the promoters might make a significant contribution to not only the pathogenesis of hormone producing adenomas, such as hypercortisolemia and PA, but also the hormonal synthesis mechanism of the normal adrenal gland. Our results might be important for the clarification of the hormonal synthesis mechanism, and the development of better treatments for hypertension due to hormone excess, such as hypercortisolemia and PA.

## Materials and Methods

### Study Patients

We studied 13 hypercortisolemia patients with CPA diagnosed between 2011 and 2015 at Kanazawa University Hospital. The diagnostic criteria for hypercortisolemia was based on the Endocrine Society Clinical Practice Guideline^2^. These guidelines recommend the measurement of late-night salivary cortisol for the diagnosis of hypercortisolemia, but this is not available in Japan. The measurement of late-night serum cortisol was used instead. We also performed ^131^I-adosterol scintigraphy as an additional evaluation method^25^. In all patients, cortisol levels were not suppressed (>49.7 nmol/L) after 1.0 mg dexamethasone treatment. Eleven patients had unsuppressed cortisol concentration at midnight and/or abnormally high urinary free cortisol. In two other patients, both the midnight serum cortisol test and the measurement of urinary cortisol concentration could not be performed, but ^131^I-adosterol scintigraphy showed unilateral uptake. All patients underwent a unilateral adrenalectomy and needed hydrocortisone replacement treatment temporarily after surgery. They discontinued the replacement from 3 to 20 months (mean 8 months). In addition, we studied 7 NFT patients (Table S2). NFT was defined as the adrenal tumor that cortisol-, aldosterone-, androgen-producing tumor and pheochromocytoma were excluded by various endocrine testing.

### Immunohistochemical analysis

Immunohistochemical staining of formalin-fixed paraffin-embedded sections of adrenal tumors was performed using monoclonal rat anti-CYP11B1 antibody and monoclonal mouse anti-CYP11B2 antibody with Chem Mate ENVISION kits (DAKO, Glostrup, Denmark) as previously reported^26^.

### Protein extraction and Western blot analysis

Protein was extracted from samples T-PER Tissue Protein Extraction Reagent (Thermo Fisher, Bremen, Germany). Western blot was performed a previously reported^27^. Samples were mixed with an SDS sample buffer (5 × buffer: 50 mM Tris-HCl (pH 6.8), 30% glycerol, 10% SDS, 250 mM dithiothreitol, 10 mM EDTA, and 0.01% Coomassie Brilliant Blue R250), subjected to SDS-10% PAGE, and transferred to a nitrocellulose membrane. The membrane was incubated with antibodies for anti-CYP11B1 antibodies (1:20,000 dilution)^11^ or GAPDH (Thermo Fisher), followed by horseradish peroxidase (HRP)-conjugated goat anti-mouse or anti-rabbit IgG (EMD Millipore). The blot was visualized using enhanced chemiluminescence reagents (PerkinElmer Life Sciences, Boston, MA) with an LAS-1000 image analyzer (Fuji Film, Tokyo, Japan).

### Gene mutation analysis

Exons 6 and 7 of the *PRKACA* gene and exons 8 and 9 of the *GNAS* gene were PCR amplified using the following primers: 5′-GTTTCTGACGGCTGGACTG-3′ and 5′-AGTCCACGGCCTTGTTGTAG-3′ for exon 6–7 of *PRKACA* ^4^, 5′-ACTATGTGCCGAGCGATCA-3′ and 5′-CAGTTGGCTTACTGGAAGTTGA-3′ for exon 8 of *GNAS*, and 5′-ACCCCAGTCCCTCTGGAATA-3′ and 5′-CCAAAGAGAGCAAAGCCAAG-3′ for exon 9 of *GNAS* ^17^. Exons 3 of the *CTNNB1* gene were PCR amplified using the following primers: 5′-GCTGATTTGATGGAGTTGGAC-3′ and 5′-CAGGACTTGGGAGGTATCCA-3′^16^. Direct sequencing was performed with an ABI PRISM 310 Genetic Analyzer (Thermo Fisher).

### Gene database of *Homo sapiens* cytochrome P450 family 11 subfamily B member 1 (*CYP11B1*)

NCBI Reference Sequence NG_007954.1 was used in this study.

### Methylation analysis by pyrosequencing

Genomic DNA was extracted from CPA, AUAT, normal ZF, and NFT flash-frozen (CPA n = 12, AUAT n = 12, normal ZF n = 4, NFT n = 4) or formalin-fixed paraffin-embedded tissue (CPA of Case 10, AUAT of Case 12, 3 normal ZF, and 3 NFT which could not be obtained as flash-frozen) using Gentra Puregene Tissue Kit (Qiagen, Hilden, Germany). We collected normal ZF samples from normal adrenal cortex with flash-frozen (n = 4) or formalin-fixed paraffin-embedded tissue (n = 3). The specimens from seven patients with NFT (n = 4), renal cell carcinoma (n = 1), and extra-adrenal paraganglioma (n = 2) were examined. We scraped the normal ZF area to extract DNA as previously reported^28^. Genomic DNA from WBC was extracted using Puregene Blood Core Kit (Qiagen). Genomic DNA from samples was treated with bisulfite and PCR amplified with primers specific for human *CYP11B1* promoter regions (Table S1). Quantitative methylation analysis of the PCR products was performed with PyroMarkGold Q96 Reagents and the PyroMarkQ24 pyrosequencing system (Qiagen).

### Cell culture and reagents

NCI H295R human adrenocortical cells were obtained from American Type Culture Collection (ATCC) and cultured in a humidified 5% CO_2_ incubator at 37 °C in DMEM/F12 medium (Sigma-Aldrich, St. Louis, MO) supplemented with 2% Ultroser G (Pall, Port Washington, NY), 1% ITS plus Premix (BD Biosciences, Bedford, MA), and 1% penicillin/streptomycin. DbcAMP was purchased from Sigma-Aldrich.

### Plasmid construction and methylation

Using genomic DNA prepared from H295R cells as a template, a 309 bp DNA fragment spanning from −302 (relative to the transcription start site) to +7 of human *CYP11B1* gene was amplified by polymerase chain reaction (PCR) with the following primers: 5′-TGGCCTAACTGGCCGGTACCCAATTCATGCCAACTCATTCC-3′ and 5′-TATCCTCGAGGCTAGCTCCAATGCTCCCTCCACCCTG-3′. The fragment was inserted into the *Kpn*I/*Nhe*I sites of a pGL4.10[luc2] vector (Promega, Madison, WI) to obtain pGL4-cyp11b1[−302/+7].

To prepare CpG-methylated plasmids, plasmid DNA was incubated with the CpG methyltransferase M.SssI (Thermo.Fisher.com). The degree of methylation of plasmid DNA was estimated by digestion with the methylation-sensitive restriction enzyme BsiEI (New England Biolabs, Hanover, MD).

### Luciferase assay

H295R cells were seeded in a collagen I-coated six-well plate. Plasmids were then transfected with FuGENE HD transfection reagent (Promega) according to the manufacturer’s protocol. Cells were lysed two days after transfection, and the luciferase activity of the lysate was measured using the Dual Luciferase Assay Reporter System (Promega).

### Real-time reverse transcription polymerase chain reaction (RT-PCR)

Total RNA was extracted from samples using ISOGEN II (Nippon Gene, Tokyo, Japan), and reverse transcribed into cDNA. Quantitative real-time PCR was then performed with the SYBR Green master mix kit (Takara, Shiga, Japan). Glyceraldehyde-3-phosphate dehydrogenase (*GAPDH*) was used as a reference gene. The primers used were 5′-GGCAGAGGCAGAGATGCTG-3′ and 5′-TCTTGGGTTAGTGTCTCCACCTG-3′ for *CYP11B1*, and 5′-GAGTCAACGGATTTGGTCGT-3′ and 5′-TTGATTTTGGAGGGATCTCG-3′ for *GAPDH* ^29^.

### Statistics and Ethics

Data are expressed as means ± SEM. Statistical analyses were performed using Excel 2016 (Microsoft, Seattle, WA) with the add-in software Statcel4 (OMS, Tokyo, Japan). Values of P < 0.05 were considered statistically significant.

This clinical study was approved by the Ethics Committees of Kanazawa University (No 2016133). The genetic modification experiment was approved by the Ethics Committees of Kanazawa University (No 2012019). Written informed consent was obtained from all patients with CPA, and from some patients with other diseases. In addition, waiver of consent of other patients was obtained from Ethics Committees of Kanazawa University. All methods were performed in accordance with the approved guidelines and regulations.

## Electronic supplementary material

Supplementary Information
