# Supplementary material for: Cortisol overproduction results from DNA methylation of CYP11B1 in hypercortisolemia
Source: Sci Rep. 2017 Sep 11;7:11205. doi: 10.1038/s41598-017-11435-2 (PMC5594008; doi:10.1038/s41598-017-11435-2)
Supplement: Supplementary file 1 — Supplementary Information [file 41598_2017_11435_MOESM1_ESM.pdf]

Supplementary Information

"Cortisol overproduction results from DNA methylation of CYP11B1 in hypercortisolemia"

Mitsuhiro Kometani[1], Takashi Yoneda[1, 2, *], Masashi Demura[3], Hiroshi Koide[4], Koshiro Nishimoto[5], Kuniaki Mukai[6], Celso E. Gomez-Sanchez[7], Tadayuki Akagi[8], Takashi Yokota[8], Shin-ichi Horike[9], Shigehiro Karashima[1], Isamu Miyamori[10], Masakazu Yamagishi[1], and Yoshiyu Takeda[1]

1 Division of Endocrinology and Hypertension, Department of Cardiovascular and Internal Medicine, Kanazawa University Graduate School of Medicine, Kanazawa, Ishikawa, 920-8640, Japan.
2 Program Management Office for Paradigms Establishing Centers for Fostering Medical Researchers of the Future, Kanazawa University, Kanazawa, Ishikawa, 920-8640, Japan.
3 Department of Hygiene, Kanazawa University Graduate School of Medicine, Kanazawa, Ishikawa, 920-8641, Japan.
4 Laboratory of Molecular and Biochemical Research, Research Support Center, Juntendo University Graduate School of Medicine, Tokyo, 113-8421, Japan.
5 Department of Uro-Oncology, Saitama Medical University International Medical Center, Hidaka, Saitama, 350-1241, Japan.
6 Department of Biochemistry and Medical Education Center, Keio University School of Medicine, Tokyo, 160-8582, Japan.
7 Endocrinology Section, G.V. (Sonny) Montgomery VA Medical Center and University of Mississippi Medical Center, Jackson, MS 39216, USA.
8 Department of Stem Cell Biology, Graduate School of Medical Sciences, Kanazawa University, Kanazawa, Ishikawa, 920-8640, Japan.
9 Advanced Science Research Center, Kanazawa University, Kanazawa, Ishikawa, 920-8640, Japan.
10 University of Fukui, Yoshida-gun, Fukui, 910-1193, Japan.
＊Corresponding author

Table S1. Characteristics of Patients with Cortisol-Producing Adenomas

Case	Age/ Gender	Cushingoid signs	BMI	HT	DM/ IGT	HL	ACTH (pg/mL)	F (nmol/L)	24h urinary F (µg/day)	Midnight F (nmol/L)	1mg DST-F (nmol/L)	Tumor size (mm)	Gene Mutation
1	44/F	(-)	19.3	(+)	(-)	(-)	6	322	ND	ND	226	19	PRKACA p.L206R
2	37/F	(+)	27.1	(+)	(+)	(+)	<5	348	93	372	361	25	PRKACA p.L206R
3	60/F	(-)	22.1	(+)	(-)	(+)	11	436	42	119	155	24	GNAS p.R201H
4	64/M	(-)	23.7	(+)	(+)	(-)	<5	441	75	149	166	30	GNAS p.R201H
5	44/F	(-)	18.4	(+)	(-)	(-)	17	521	36	127	279	20	GNAS p.R201H
6	58/F	(-)	20.4	(+)	(-)	(-)	<5	212	35	348	102	13	GNAS p.R201S
7	52/M	(+)	32.5	(+)	(-)	(-)	11	480	136	530	571	27	GNAS p.R201C
8	58/F	(-)	26.6	(+)	(-)	(-)	6	223	31	138	135	22	GNAS p.Q227R
9	61/F	(-)	20.7	(+)	(-)	(-)	<5	430	44	160	83	30	WT
10	33/F	(-)	29.6	(+)	(-)	(-)	<5	221	31	243	259	23	WT
11	55/M	(-)	21.1	(-)	(-)	(-)	16	428	40	179	83	30	WT
12	53/F	(-)	22.1	(+)	(+)	(-)	<5	417	70	579	643	23	WT
13	65/M	(-)	30.5	(+)	(-)	(+)	5	221	ND	ND	69	45	WT

BMI, body mass index; HT, hypertension; DM, diabetes mellitus; IGT, impaired glucose tolerance; HL, hyperlipidemia; F, serum cortisol concentration; DST, dexamethasone suppression test; ND, no data; WT, wild type for both *PRKACA* and *GNAS* genes. '(+)' represents positive, and '(-)' represents negative.

Table S2. Characteristics of Patients with Non-Functioning Adrenal Tumor

Case	Age/ Gender	Cushingoid signs	BMI	HT	DM/ IGT	HL	ACTH (pg/mL)	F (µg/dL)	24h urinary F (µg/day)	Midnight F (µg/dL)	1mg DST-F (µg/dL)	Tumor size (mm)	Final Diagnosis
14	61/F	(-)	20.2	(-)	(-)	(-)	23	11	14	4	1	11	NFT
15	57/F	(-)	21.4	(-)	(-)	(-)	25	15	35	2	1	32	NFT
16	40/M	(-)	22.0	(-)	(-)	(-)	10	12	ND	ND	1	28	NFT
17	24/M	(-)	18.8	(-)	(-)	(-)	29	14	31	5	1	45	NFT
18	78/F	(-)	22.4	(-)	(-)	(-)	8	14	37	6	1	37	NFT
19	62/M	(-)	32.5	(+)	(-)	(+)	11	11	60	5	1	21	NFT
20	44/F	(-)	19.7	(-)	(-)	(-)	24	6	64	2	0.2	160	NFT

NFT, non-functioning adrenal tumor; BMI, body mass index; HT, hypertension; DM, diabetes mellitus; IGT, impaired glucose tolerance; HL, hyperlipidemia; F, serum cortisol concentration; DST, dexamethasone suppression test; ND, no data. ‘(+)’ represents positive, and ‘(-)’ represents negative.

Table S3. Primers used for pyrosequencing

Primer	Sequence (5’ to 3’)
CYP11B1 Pyro-F 1-2	TTGTAATTTTTTTATTTTGTTTGGTGTTT
CYP11B1 Pyro-R 1-2	ATACACCCCCAATAAATCCCTAC
CYP11B1 Pyro-S1	TGTTTGGTGTTTTGTTTT
CYP11B1 Pyro-S2	TGGTTTTGGATTTGTTTGAG
CYP11B1 Pyro-F3	AGGTTAGGGTTGGAGGTAGG
CYP11B1 Pyro-R3	AACCCCATCCATCTTACTCCTC
CYP11B1 Pyro-S3	ATTGGGGGTGTATGA
CYP11B1 Pyro-F4	GGATGGGGTTTTTATTTTATTTAAGAGT
CYP11B1 Pyro-R4	CCCAATAATCATTCAAAAACAAATTACTCA
CYP11B1 Pyro-S4	ATTTATTTTTTTGTAAGGTTTATA

‘F’, ‘R’ and ‘S’ indicate forward, reverse and sequence primers, respectively. The 5' ends of reverse primers are biotinylated.

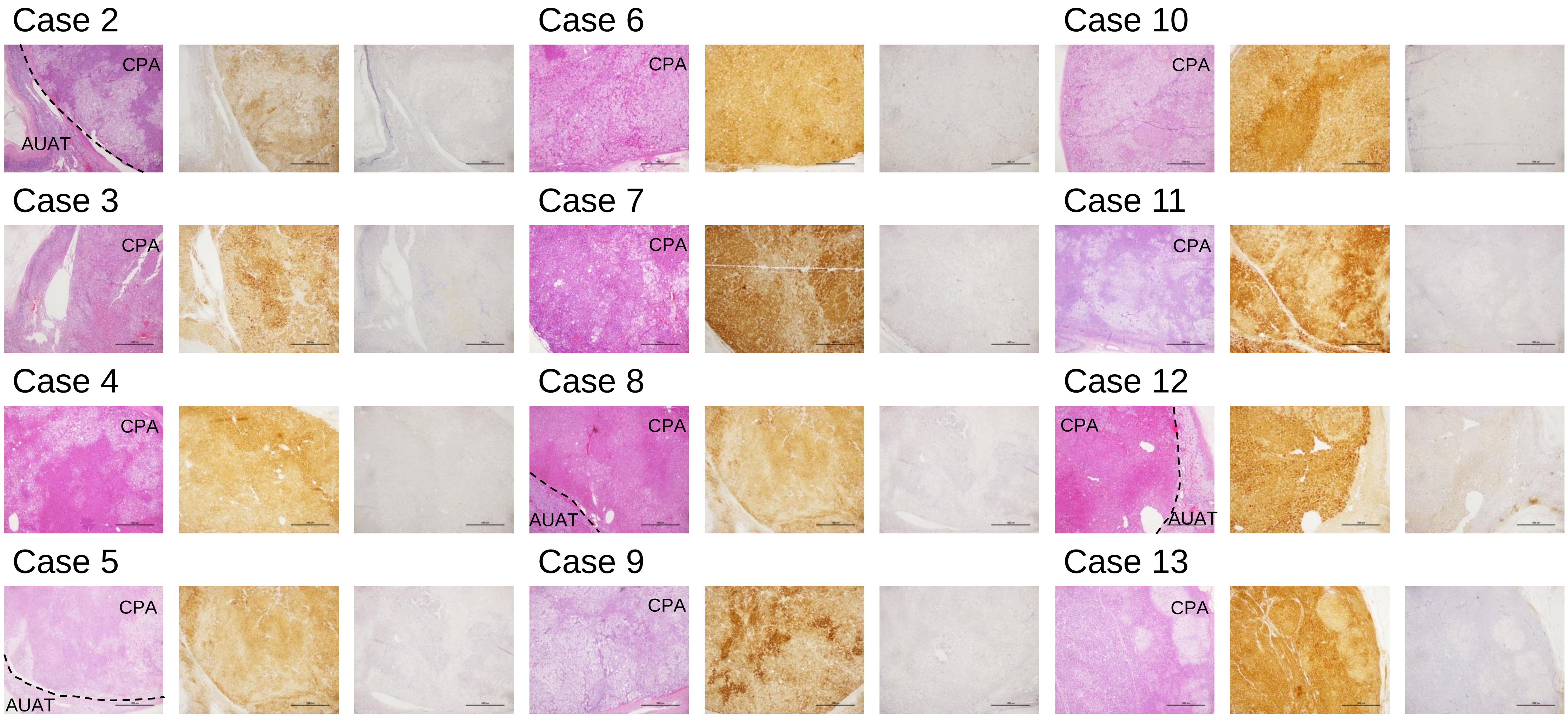

Figure S1. Immunohistochemical analysis of *CYP11B1* overexpression in all cases of cortisol-producing adenomas

Formalin-fixed paraffin-embedded tissue sections were stained with Hematoxylin & Eosin (HE) (left), anti-CYP11B1 (middle), and anti-CYP11B2 antibodies (right). CPA, cortisol-producing adenoma; AUAT, adjacent unaffected adrenal tissue.

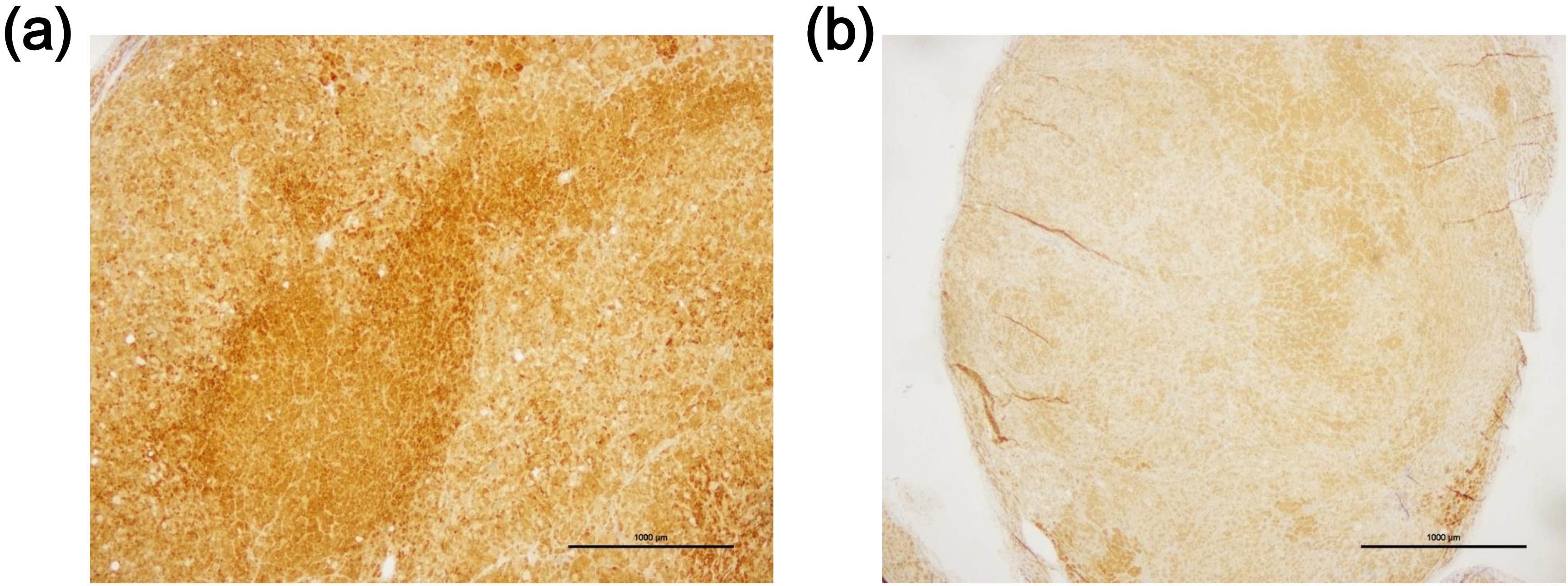

Figure S2. Two cortisol-producing adenomas with atypical CYP11B1 staining patterns (case 10)
a, The tumor with stronger staining. **b**, The tumor with weaker staining.

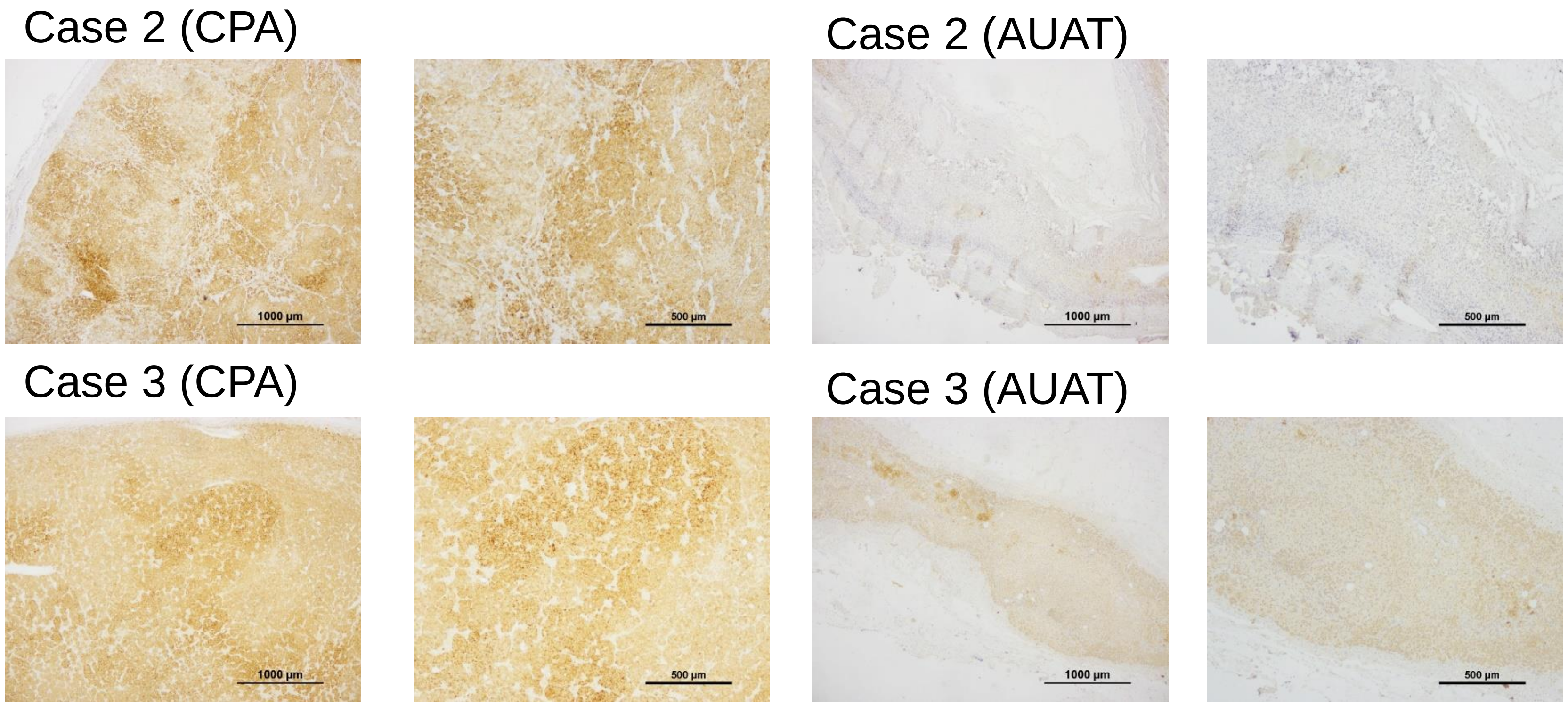

Figure S3. Immunohistochemical analysis of *CYP11B1* expression in cortisol-producing adenomas and adjacent unaffected adrenal tissue

Formalin-fixed paraffin-embedded tissue sections were stained with anti-CYP11B1. *CYP11B1* expression in CPAs was higher than AUATs in both cases.

CPA, cortisol-producing adenoma; AUAT, adjacent unaffected adrenal tissue.

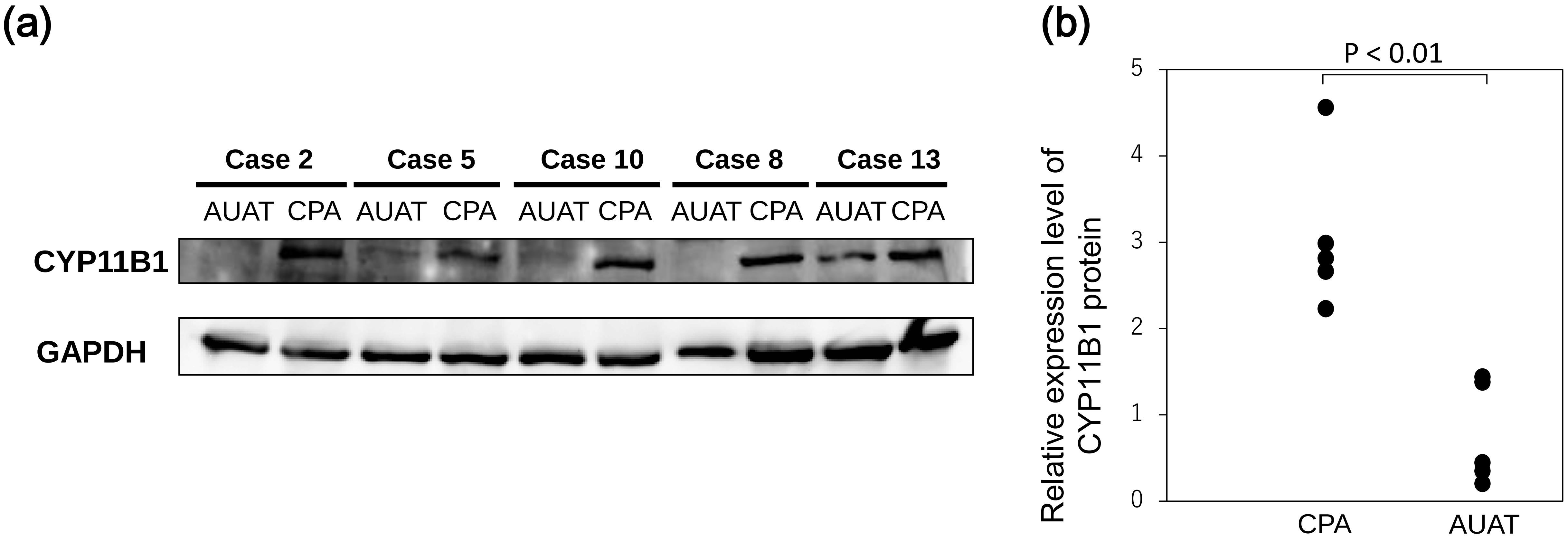

Figure S4. Expression of CYP11B1 protein in cortisol-producing adenoma
a, Western blot analysis of tissue homogenates (20 µg of protein) by using anti-CYP11B1 and anti-GAPDH antibodies. Full-length blots are shown in Figure S4c.
AUAT, adjacent unaffected adrenal tissue ; CPA, cortisol-producing adenoma.
b, The density of each band was measured by LAS-1000 image analyzer, and data were normalized to GAPDH. The x-axis represents the tissue sample category: CPA (n = 5) and AUAT (n = 5). The mean of AUAT was set to 1.0. Significant difference was evaluated by the Mann-Whitney U test.

(c)

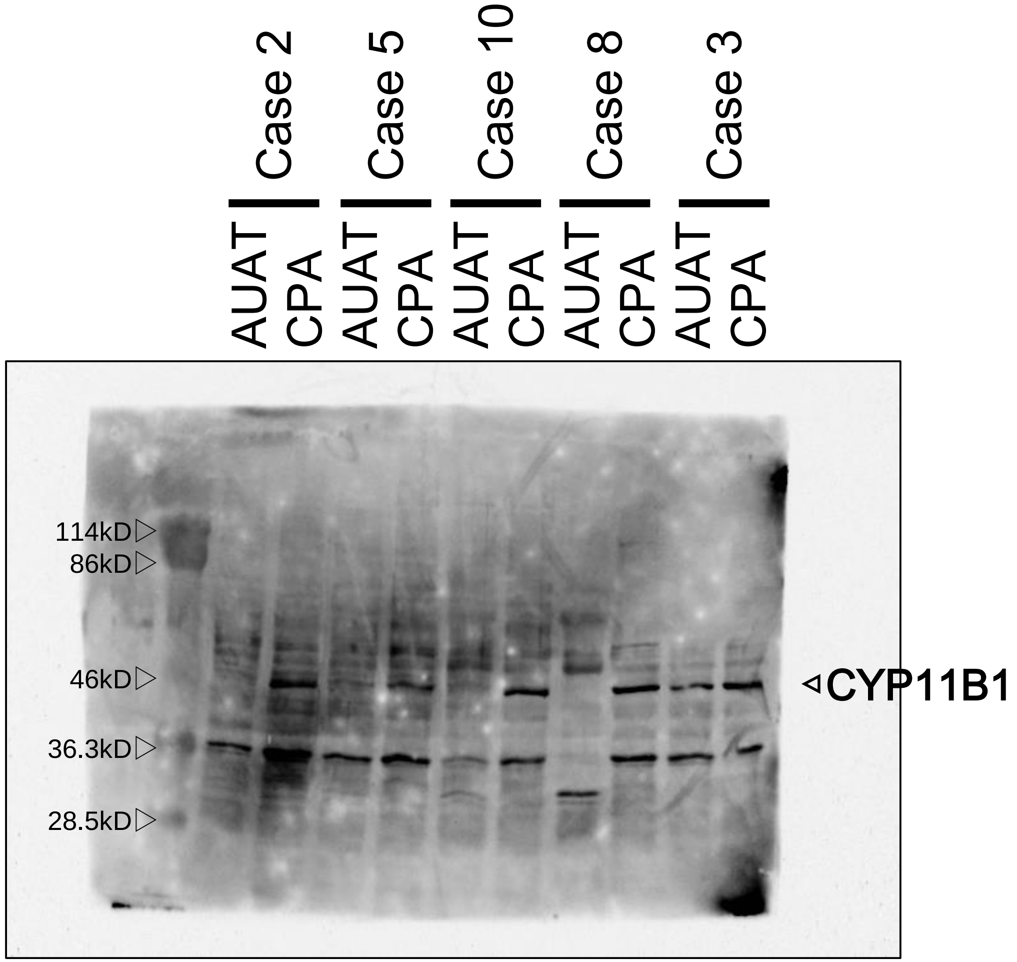

CYP11B1

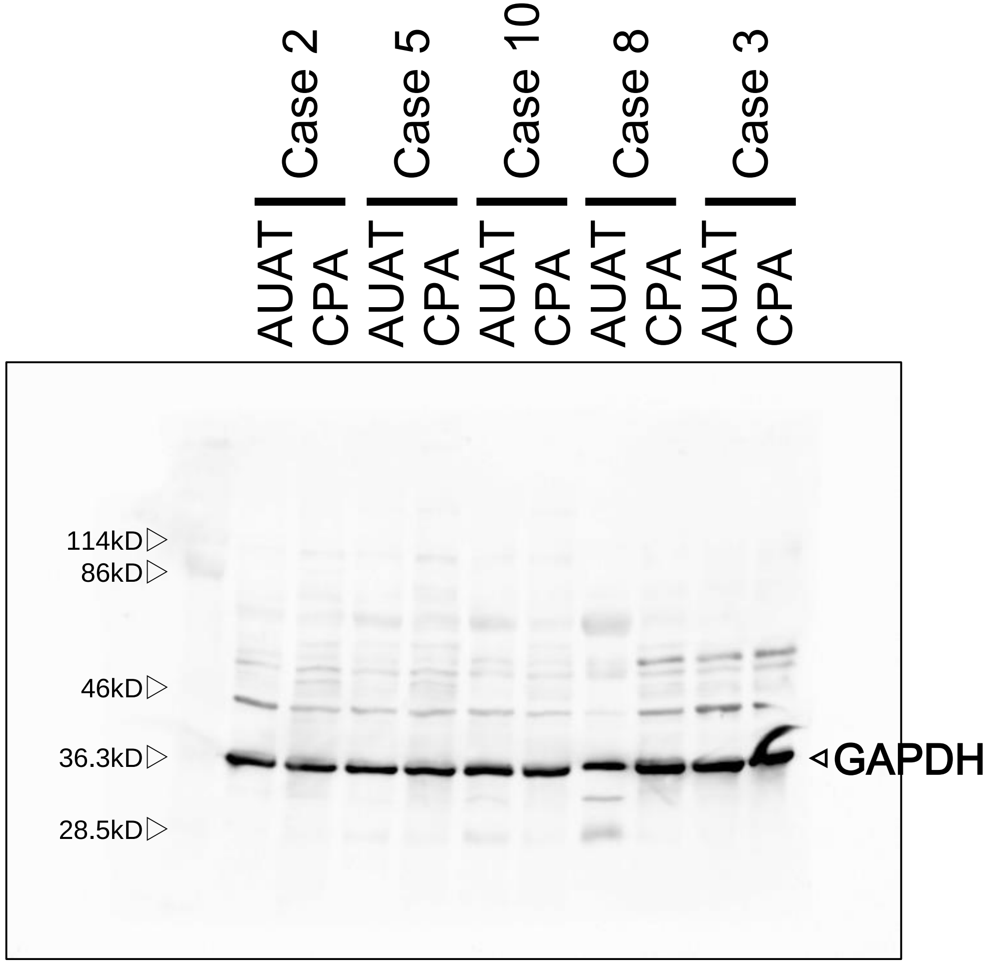

GAPDH

Figure S4. Expression of CYP11B1 protein in cortisol-producing adenoma
c, Full-length blots of CYP11B1 (left panel) and GAPDH (right panel) are shown.

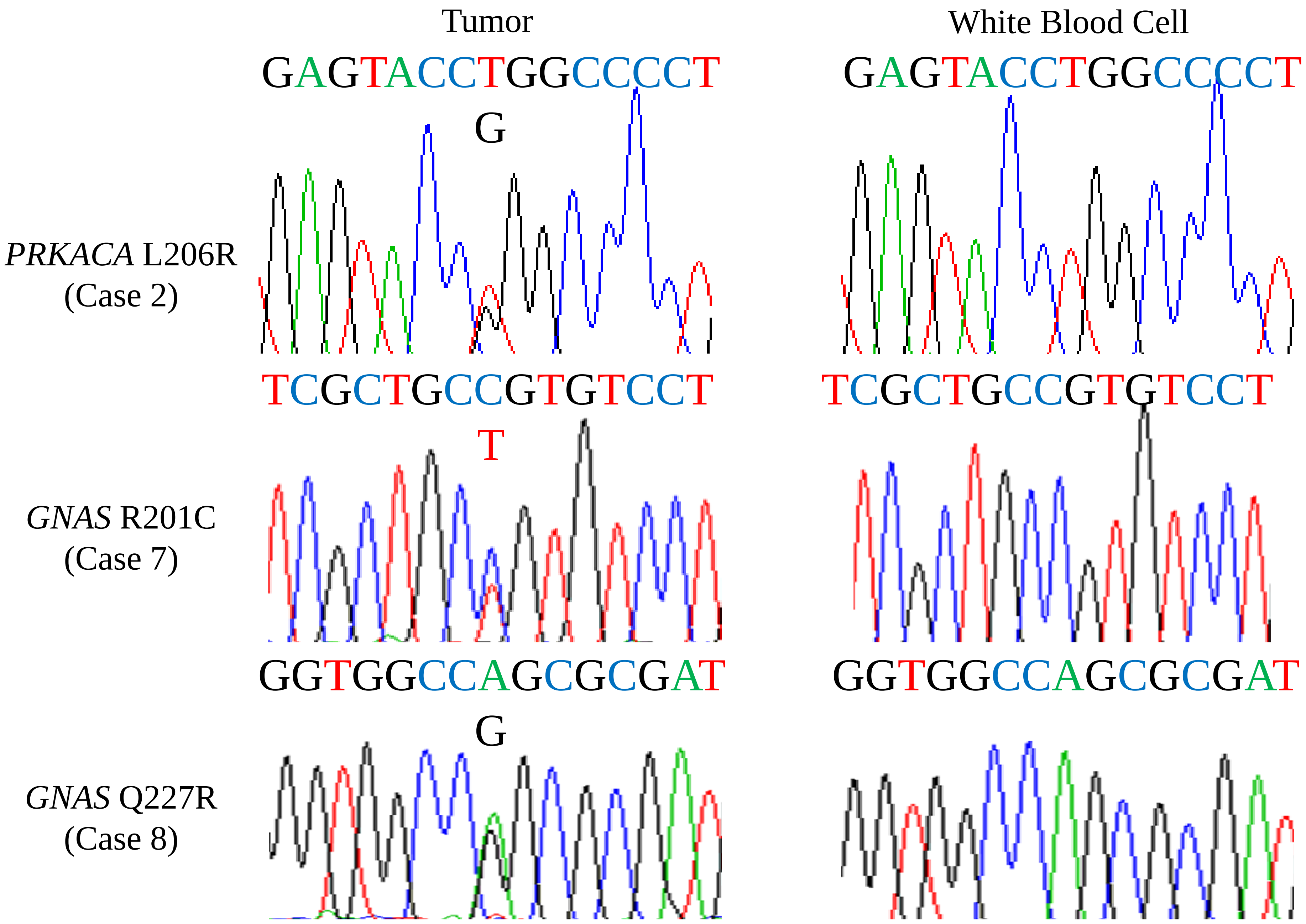

Figure S5. Gene mutation analysis of *PRKACA* and *GNAS* gene

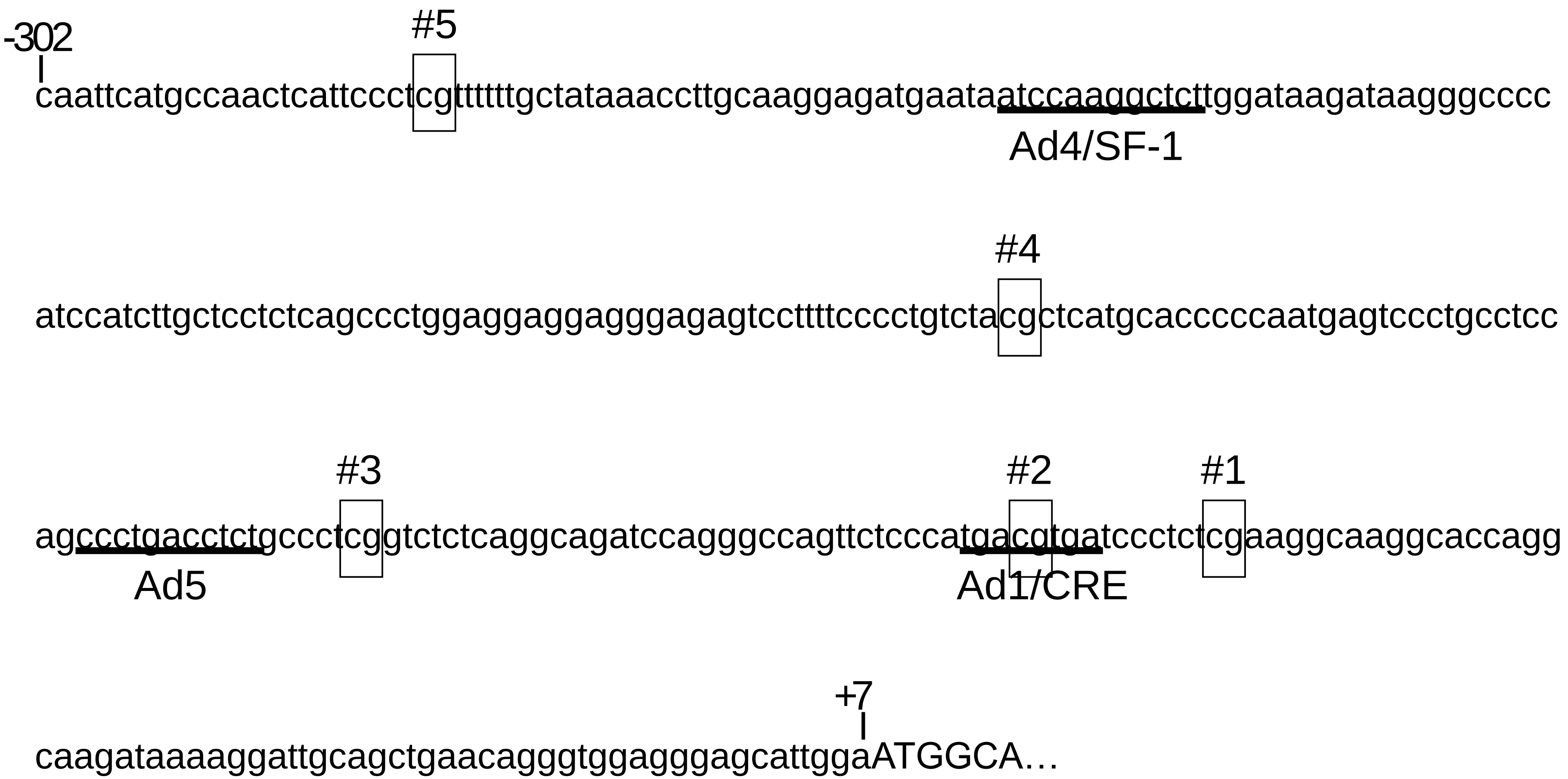

Figure S6. Oligonucleotide sequence of the DNA fragment spanning -302 (relative to the transcription start site) to +7 of the human *CYP11B1* gene

Transcription factor-binding sites are indicated with underlines, and each open square represents one CpG site. Note that the start codon of CYP11B1 is located at +8 to +10.

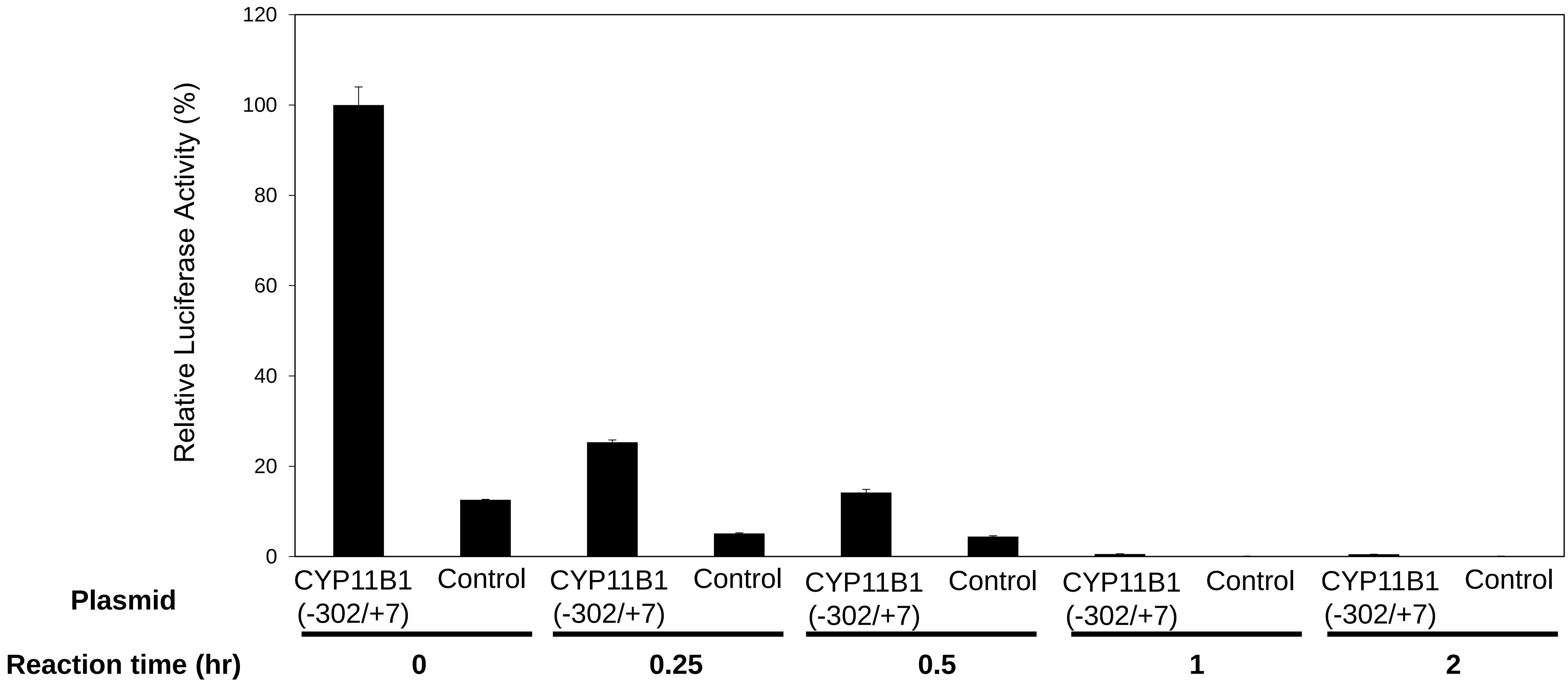

Figure S7. Effect of methylation on luciferase activity of reporter plasmids
Both pGL4.10[luc2](control) and pGL4-cyp11b1[-302/+7](CYP11B1[-302/+7]) were incubated with the CpG methyltransferase M.SssI for the indicated period and subject to Luciferase assay. Note here that although the luciferase activity of the control plasmid was reduced by methylation, the effect of methylation was smaller when compared with pGL4-cyp11b1[-302/+7]. Luciferase activity of the unmethylated CYP11B1[-302/+7]) sample (reaction time = 0 h) was set to 100%, and the mean ± SEM (n = 4) of the data are shown.

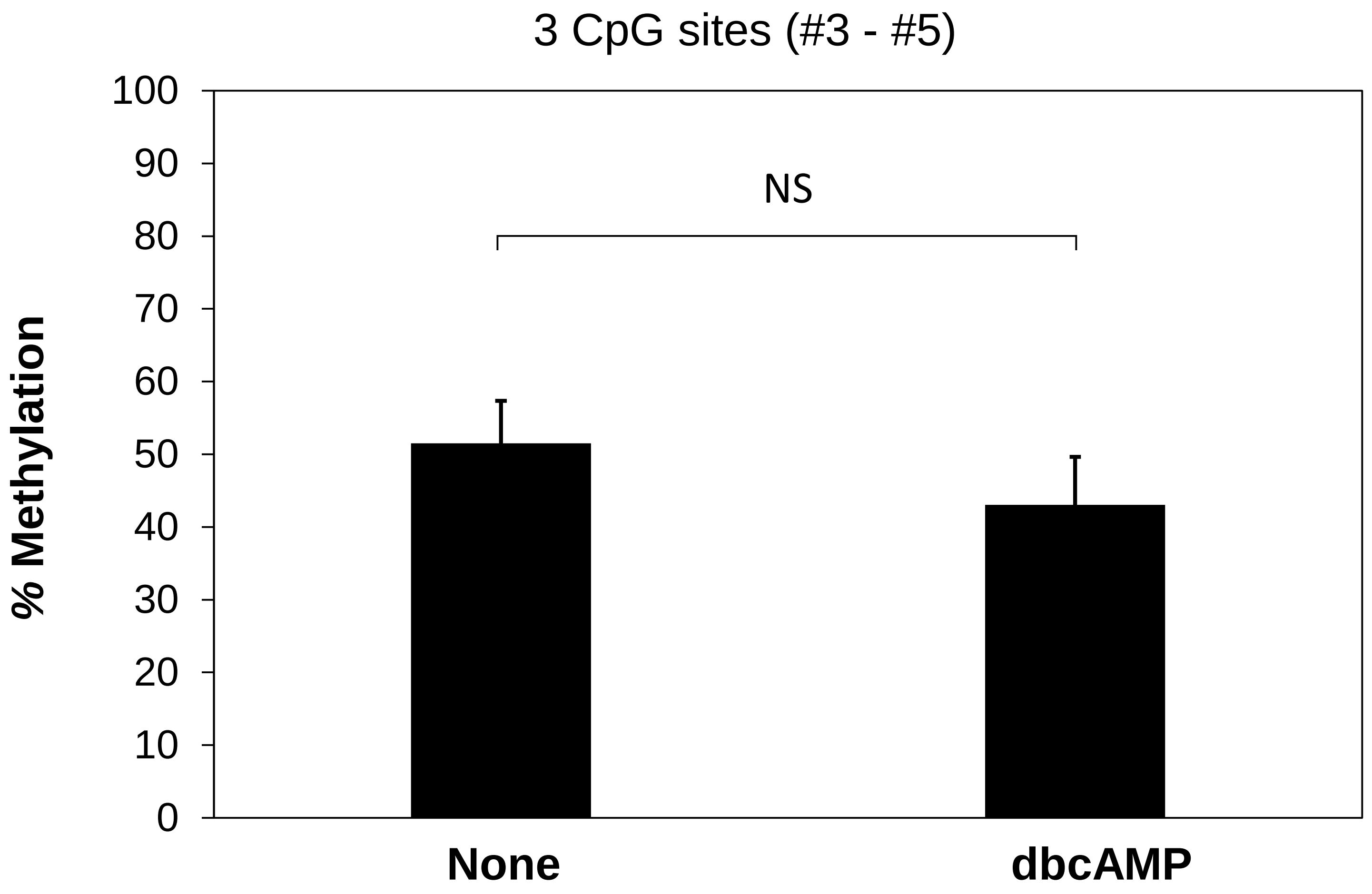

Figure S8. Effect of dbcAMP treatment on DNA methylation at CpG#3-5 of the *CYP11B1* promoter
After stimulation with dbcAMP, methylation levels at 3 CpG sites (#3 - #5) in the *CYP11B1* promoter was measured by pyrosequencing.
Data are shown as the mean ± SEM (n=3), and analyzed by the Mann-Whitney U test.
NS, not significant.

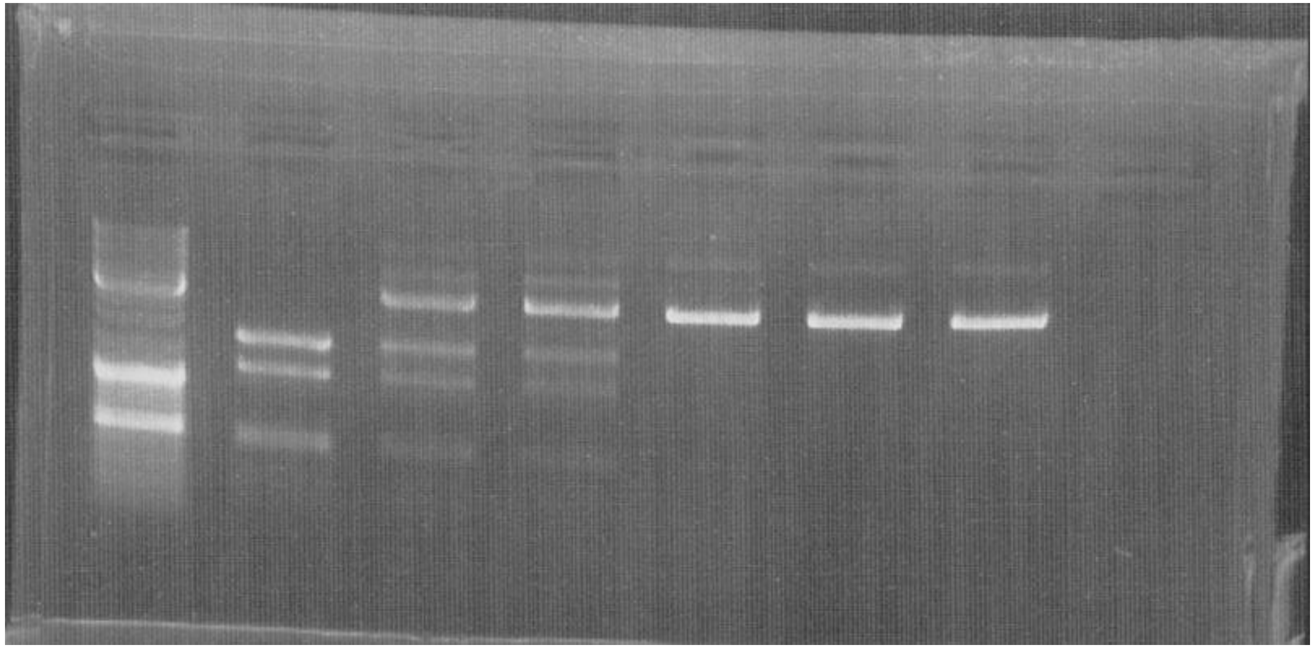

Figure S9. Confirmation of plasmid methylation
Full-length gel of Figure 4b is shown.
